# High-resolution nanomechanical analysis of suspended electrospun silk fibers with the torsional harmonic atomic force microscope

**DOI:** 10.3762/bjnano.4.25

**Published:** 2013-04-05

**Authors:** Mark Cronin-Golomb, Ozgur Sahin

**Affiliations:** 1 Department of Biomedical Engineering, Tufts University, Medford, MA 02155, USA; 2 Rowland Institute at Harvard, Harvard University, Cambridge, MA 02142, USA; 3 presently with Department of Biological Sciences and Department of Physics, Columbia University, New York, NY 10027, USA

**Keywords:** atomic force microscopy, nanomechanical characterization, silk fibers, tissue scaffolds, torsional harmonic cantilevers

## Abstract

Atomic force microscopes have become indispensable tools for mechanical characterization of nanoscale and submicron structures. However, materials with complex geometries, such as electrospun fiber networks used for tissue scaffolds, still pose challenges due to the influence of tension and bending modulus on the response of the suspended structures. Here we report mechanical measurements on electrospun silk fibers with various treatments that allow discriminating among the different mechanisms that determine the mechanical behavior of these complex structures. In particular we were able to identify the role of tension and boundary conditions (pinned versus clamped) in determining the mechanical response of electrospun silk fibers. Our findings show that high-resolution mechanical imaging with torsional harmonic atomic force microscopy provides a reliable method to investigate the mechanics of materials with complex geometries.

## Introduction

Dynamic atomic force microscopy (AFM) methods provide opportunities for high-resolution compositional mapping of heterogeneous samples [1]. Recent developments in dynamic AFM methods offer the possibility of relating the measured vibration signals to the particular physical properties of the samples, such as elastic modulus, viscosity, adhesion, and chemical affinity [2–16]. These developments are accomplished by employing multiple excitation and detection frequencies during dynamic AFM imaging [17–26]. A critical element of these mechanical measurements is the physical model being used to relate the force–distance curves to parameters representing the material properties. Although contact-mechanics models can be used for a wide variety of polymer composites, block-copolymers, and biomaterials [27–34], these models are not applicable to materials with complex geometries. For example, the use of local interaction models provides limited information in the case of suspended structures where bending modulus, geometry, and mechanical tension are the key determinants of tip–sample interactions. The interpretation of measurements on these kinds of samples requires simultaneous analysis of mechanical measurements and topography, as well as comparison of various mechanical models.

In this work we investigate the mechanical behavior of electrospun silk fibers, which are used for making scaffolds for bone-tissue engineering [35]. Mechanical characteristics of these structures are important because their primary purpose is to mimic extracellular-matrix conditions, including their rigidity. Bulk properties, while important, are not sufficient to predict the mechanical behavior of the electrospun fibers. Geometry of the network of fibers, fiber diameter, mechanical boundary conditions at the nodes of the fiber network (pinned versus clamped), and the presence of mechanical tension within the fibers can influence their mechanical behavior. We have carried out experiments to determine the relative influences of these parameters on the mechanics of electrospun silk fibers.

## Results and Discussion

**Figure 1:**
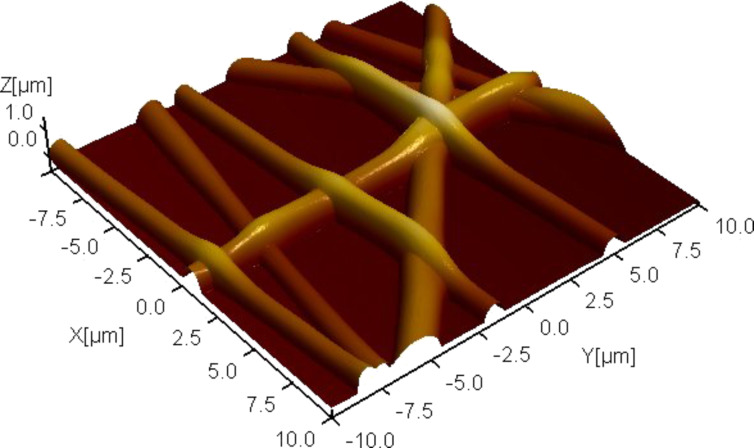
Topography of electrospun silk fibers on a glass substrate. The 3-D image is rendered according to the local height measured by the atomic force microscope. The scan size is 20 × 20 µm^2^. The fibers form a mesh-like network. Branches between intersections occasionally form suspended fibers, allowing us to investigate their mechanical behavior.

Electrospun silk fibers form mesh-like networks with nodes and branches. Diameters of these fibers are typically in the submicron range. Separation between the nodes, defined by intersections between two or more fibers, can be on the order of one to ten micrometers depending on the electrospinning process. This size scale is readily accessible by atomic force microscopy for topographical and mechanical characterization. When several fiber layers are deposited to form fibrous tissue scaffolds, these branches form suspended structures. We have limited our experiments to samples that are formed by two to three layers of fibers so that we can readily identify individual fibers. Although some of the branches in the first few layers appear to rest on the substrate, some branches still form suspended fibers sufficient for our study (Figure 1).

We have used torsional harmonic AFM to determine the surface topography and local mechanical response with high spatial resolution [20,31]. This mode uses a T-shaped cantilever with an offset tip. When used in dynamic AFM, the cantilever vibrates up and down, similar to conventional cantilevers. In addition to the vertical motion, tip–sample-interaction forces twist the cantilever by a detectible amount. The high bandwidth of torsional motion allows accessing higher harmonics of the tip–sample-interaction forces to reconstruct tip–sample-force waveforms. This process involves calibration of the frequency response of the torsional mode by measuring its resonance frequency and quality factor (either by frequency sweeps or from the thermal peak in the noise spectrum). The gain of the torsional mode, defined as the photodetector signal corresponding to a unit amount of a quasi-static force acting on the tip, is determined by independently measuring the quasi-static force from vertical deflections while monitoring the torsional deflection signal. Note that the same force acts on both vertical and torsional modes. Therefore, after calibrating the vertical spring constant, the gain of the torsional mode can be determined by comparing time-average detector signals in vertical and horizontal channels during a tapping-mode AFM experiment. To minimize contributions of drift in quasi-static deflection signals, we previously developed a procedure that takes advantage of the transitions between attractive and repulsive modes [36]. The calibrated frequency response and gain of the torsional mode allows the reconstruction of the tip–sample-force waveforms. A computer program carries out these calculations in real time during the tapping-mode imaging process. The program also corrects for nonlinearities of the position-sensitive diode and for crosstalk from large vertical signals into torsional vibration signals. Once the tip–sample-force waveform is determined, the program constructs force–distance curves using the distance information in the vertical deflection signals [20]. It is possible to analyze these force–distance curves according to various physical models to obtain parameters describing the mechanical response of the sample. In the case of electrospun silk fibers, we have calculated both the local elastic modulus and the local spring constant values. The elastic modulus is calculated according to the Derjaguin–Muller–Toporov (DMT) model and the spring constant (stiffness) is calculated by fitting the unloading portion of the force–distance curve with a straight line. For the DMT model, we used a tip radius of 7 nm, which is characterized by blind reconstruction from a sample with sharp edges. Our calculations assumed that the unloading portion of the force distance-curve is the region between the peak force and the point where the force drops to 20% of the peak value.

Initially, we calculated both the elastic modulus and spring constant values regardless of their appropriateness for describing local mechanical response. We identified the appropriate parameter by analyzing the simultaneously obtained topography image. In regions where silk fibers appear to rest on the substrate, elastic modulus values are considered to be the relevant parameter. In regions where the silk fibers appear to be suspended, spring constant values are considered to be the relevant parameter. In the latter case, we further analyzed whether the measured spring constant values across the suspended fiber can be predicted by various mechanical models.

**Figure 2:**
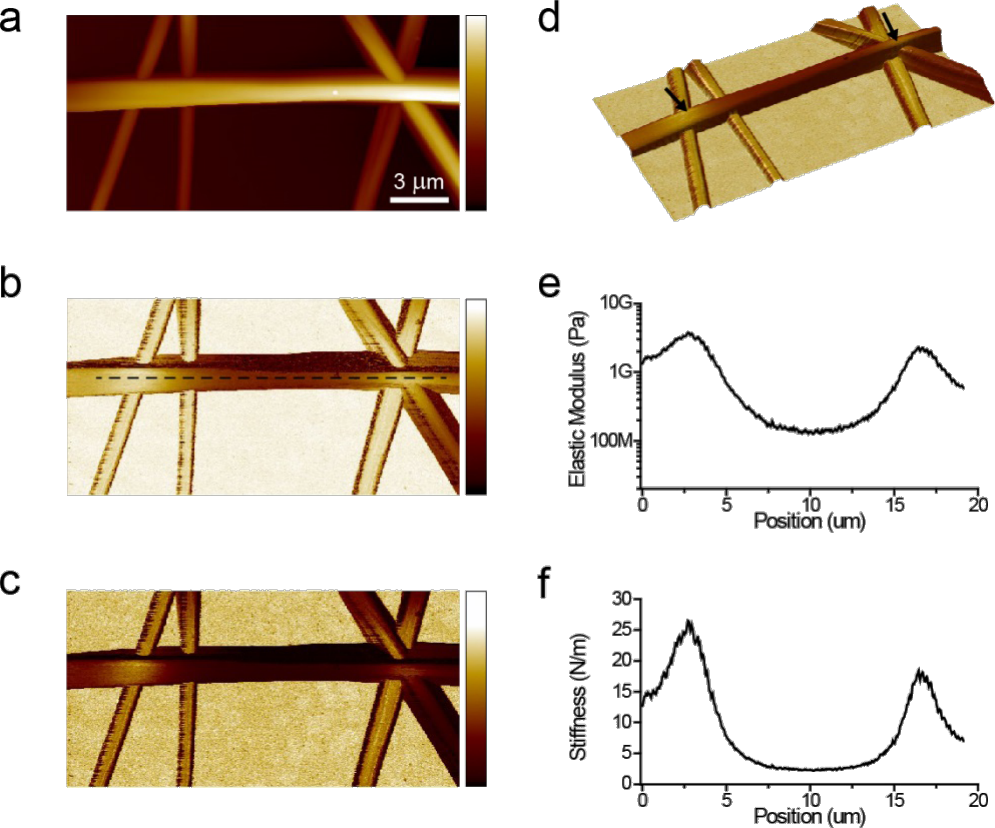
Simultaneously measured topography (a), elastic modulus (b), and stiffness (c) maps obtained from electrospun silk fibers. Color bars in (a–c) correspond to the ranges in height (0–1.8 µm), elastic modulus (10 MPa to 10 GPa, mapped logarithmically), and stiffness (0–5 N/m). The horizontal fiber appears to be suspended above the glass substrate. A 3-D rendering of the topography image is given in (d). The fiber is suspended between positions indicated by arrows in (d). This image is colored according to the local spring constant. Both the elastic modulus and stiffness maps show gradual variations across the suspended silk fiber. Line profiles of elastic modulus and stiffness across the dashed line in (b) are given in (e) and (f), respectively. While the local elastic modulus of the silk fiber is likely to be constant across the length of the fiber, the values in (e) show significant variation. This is because the elastic modulus values in (b,e) are calculated by the DMT contact-mechanics model, which does not take the suspended geometry of the fiber into account. Therefore, the regions of the elastic modulus image corresponding to suspended fibers are not reliable. These regions are better analyzed in the light of mechanical models describing the entire suspended structure by using the stiffness values in (c,f).

Figure 2 shows topography, elastic modulus, and stiffness images obtained from electrospun silk fibers. The sample was prepared from pure silk electrospun at 8 kV over a distance of 7 inches at pH 8. A suspended fiber branch extending from left to right is identified based on its height relative to the remaining fibers and the glass substrate. Two fibers extending from top to bottom on the left side of the image appear to be resting on the glass substrate, whereas the fibers on the right side intersect with each other and the suspended horizontal fiber at approximately the same location. The fibers that are resting on the substrate exhibit a uniform elastic-modulus profile (about 5 GPa); however the suspended horizontal fiber exhibits a gradual decrease towards its midpoint. The stiffness map and its corresponding line profile show similar trends, indicating qualitative agreement between the two parameters. Because the model used for calculating the elastic modulus does not account for the suspended geometry of the fiber, we rely on the spring constant values to understand the mechanism responsible for the observed mechanical response.

It is also important to consider the possible effect of the inertia of the fibers on the measured forces. The inertia of the fibers can be neglected if the resonance frequency of the suspended silk fibers is much higher than the frequency of cantilever vibrations analyzed in the experiment. Torsional harmonic AFM relies on force measurements at frequencies up to the torsional resonance frequency of the cantilever, about 1 MHz. One can estimate the resonance frequency of the suspended fiber structure using Euler–Bernoulli analysis [37]:

[1]
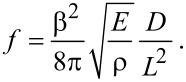


Here the constant β^2^ is equal to 22.373 for the clamped-end boundary condition. *E* and ρ are the elastic modulus and mass density, and *D* and *L* are the diameter and length of the silk fiber. Using *E* = 10 GPa, ρ = 1.3 g/cm^3^, *D* = 2*R* = 0.52 µm, we obtain the resonance frequency ƒ = 129.4 MHz, which is far above the torsional resonance frequency of the AFM cantilever. Therefore, we neglect the effects of the inertia of the fibers in our experiments.

**Figure 3:**
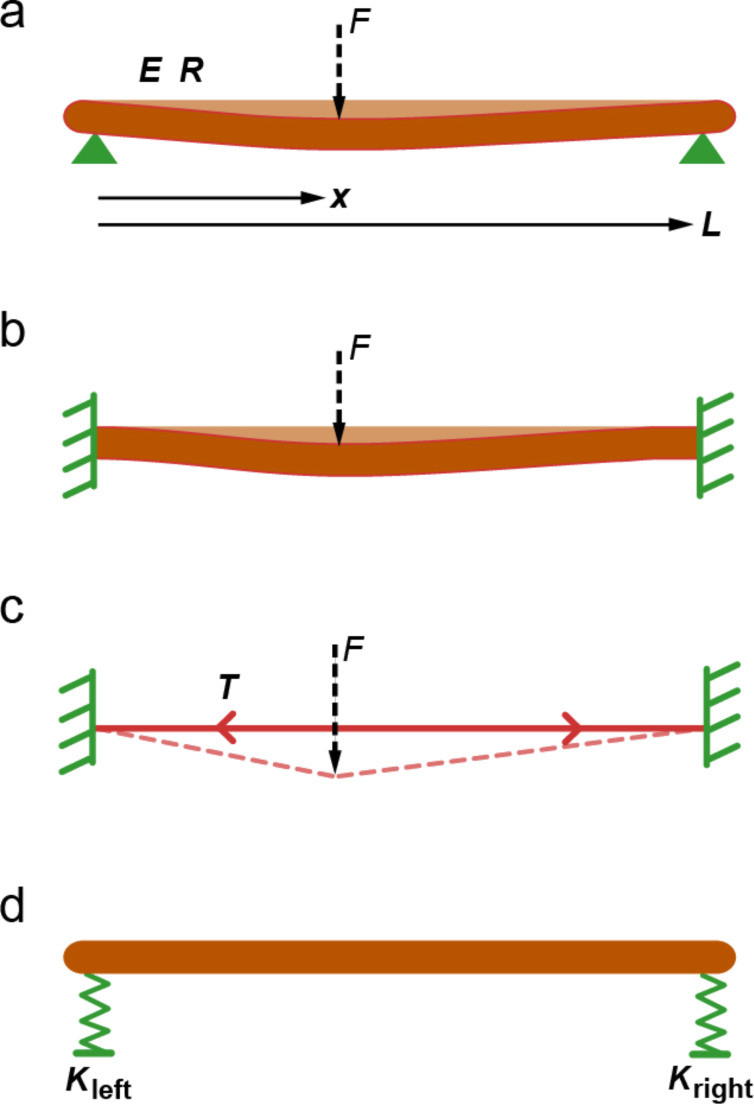
Illustration of possible mechanisms determining the local spring constant of suspended silk fibers. (a) “Pinned-end model” assumes that the fiber displacement is zero at the nodes; however, there is no constraint on its angle at the nodes. *E* elastic modulus, *R* is fiber radius, *L* is branch length. (b) “Clamped-end model” assumes that both the angle and displacement at the nodes are zero. (c) “Tension model” assumes the fiber has a built in tension *T* and negligible bending modulus. (d) “Suspended rigid rod model” assumes the nodes have finite spring constants *K*_left_ and *K*_right_. Note that the spring constant according to the mechanism in (d) acts in series with the mechanisms in (a–c).

While gradual changes in stiffness of the suspended fiber are not surprising, the precise mechanism that determinines the mechanical response of the suspended fiber is not immediately clear. We identified three scenarios that can qualitatively explain the observed results. The suspended fiber can be viewed as a cantilever structure pinned at both ends, clamped at both ends, or as a string that is under tension. Graphical depictions of these three cases are given in Figure 3a–c. All three scenarios would result in variations in the local stiffness of the fiber as probed by the AFM tip. However, the stiffness values predicted by these models would have different spatial dependencies. It is worth noting that all these models assume that the displacement of the fiber at the nodes is zero, which would result in an effectively infinite spring constant at these locations. In our experiments, the spring constant at the nodes are finite and determined by both the tip–fiber contact-mechanics and the spring constant associated with fiber–fiber interactions at the nodes. To take these effects into account, we assumed a simple model depicted in Figure 3d, which we refer to as the suspended-rigid-rod model. The variables required by all four models and the equations decribing local spring constants based on these models are listed in Table 1. Note that the effective spring constant orignating from the suspended-rigid-rod model acts in series with the other three models. Additionally, a more sophisticated model could include the fiber–tip spring constant, which acts in series with the spring constants due to fiber–fiber interactions at the nodes. The two models have to give the same total spring constant at the nodes, but the model in Figure 3d results in a linear dependancy to the distance from the nodes and the model that takes the fiber–tip spring constant into account results in a nonlinear dependancy on the distance.

**Table 1:** Description of the variables and equations for spring constants based on the four mechanical models depicted in Figure 3. The values of variables calculated by curve fitting are given in the last column. Standard errors are given in parenthesis with the same units.

Model description	Variables	Constants	Equation	Best fit (standard error)

Pinned end	*E*: elastic modulus *L*: branch length *x*: position	*R*: fiber radius	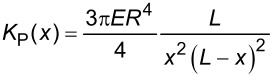	*E* = 35.47 GPa (0.29) *L* = 13.59 µm (0.014)
Clamped end	*E*: elastic modulus *L*: branch length *x*: position	*R*: fiber radius	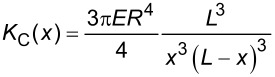	*E* = 10.16 GPa (0.13) *L* = 14.94 µm (0.021)
Tension	*T*: tension *L*: branch length *x*: position		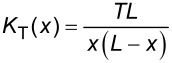	T = 16.73 µN (0.42) *L* = 13.47 µm (0.003)
Suspended rigid rod	*L*: branch length *x*: position	*K*_left_: left spring constant *K*_right_: right spring constant	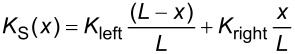	

**Figure 4:**
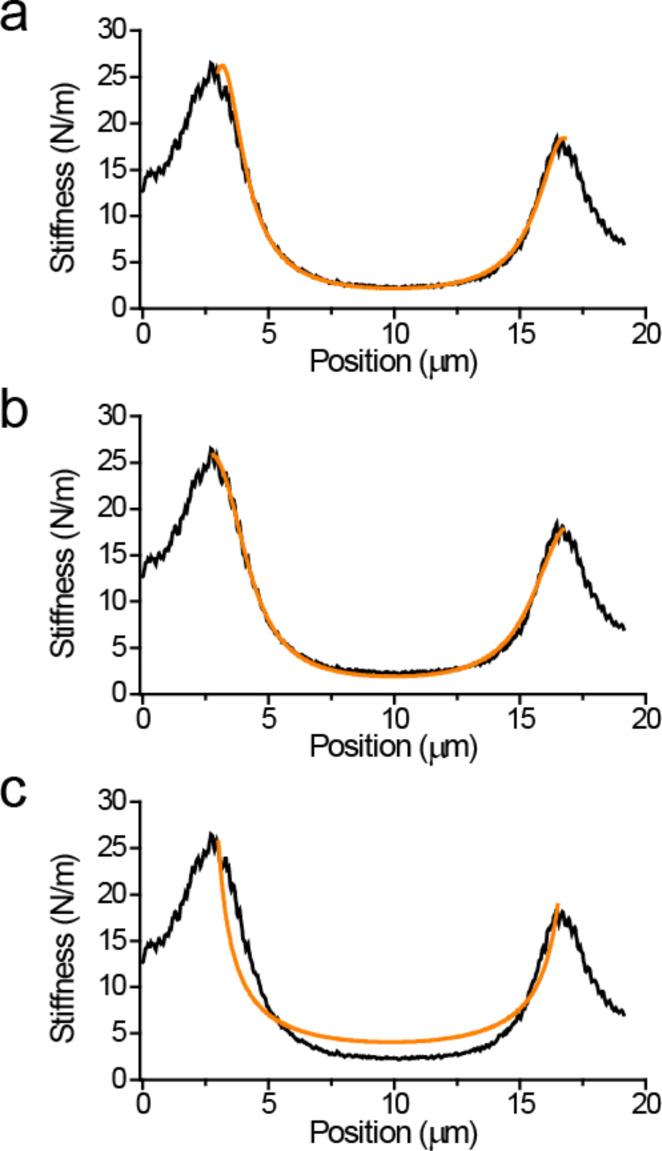
Curves described by equations for pinned end (a), clamped end (b), and tension (c) models fitted to the data. Values of the variables used for fitting are listed in Table 1.

To determine if any of the three models in Figure 3a–c, in combination with the rigid-rod model in Figure 3d, can explain the observed variations in spring constant, we attempted to fit the data in Figure 2f with the total spring constant *K*(*x*) based on equations listed in Table 1. For the pinned-end and clamped-end models, we used *E* and *L* as variables for the fitting. For the tension model, we used *T* and *L* as variables. For all three models, *K*_S_ and *K*_T_ are assumed to be constant and equal to 26.2 N/m and 18.4 N/m, respectively. In addition we used *R* = 0.26 µm. The values for *K*_S_ and *K*_T_ are determined from the peak spring constant values in the data plotted in Figure 2f. The value for *R* is determined from the topography measurements in Figure 2a. The values of the parameters used for fitting are also listed in Table 1 and the resulting curves are plotted in Figure 4.

From the results of the fitting procedures we see that all three models could reproduce qualitative trends similar to the measured spring-constant profile. However, the tension model did not produce a good overall fit to the data. The tension value calculated as 16.7 µN translates to a tensile stress of ≈78.6 MPa, which is a relatively high value, but silk could potentially sustain such large stresses. The other two models provided a better overall fit to the data as seen in Figure 4a and Figure 4b. However, quantitatively the pinned-end model required *E* to be 35.5 GPa, which is higher than even in native silk fibers (*E* ≈ 14 GPa [38]). The clamped-end model predicts *E* to be around 10.2 GPa and therefore it is more likely that this model provides a better description of suspended silk fibers. Note that the numerical estimates depend on the fourth power of the fiber radius; however, even with 10% increase in radius, the pinned end model cannot provide an elastic modulus value that falls in a plausible range (<15 GPa).

## Conclusion

In this paper we have investigated the mechanical behavior of electrospun silk fibers whose geometry does not allow the straightforward use of contact-mechanics models. We used elastic-modulus and stiffness maps determined by torsional harmonic AFM and fitted the data obtained from suspended silk fibers with models that could potentially explain the observed variations in stiffness. This analysis revealed that a clamped-end model, in which the displacements and bending of a fiber are restricted at nodes, successfully describes the observed characteristics. We expect that the applications of the general methodology used in this paper could also be extended to characterization of cytoskeletal protein networks and microelectromechanical (MEMS) devices where suspended structures are commonly encountered.

## References

[1] García R, Magerle R, Perez R (2007). Nat Mater.

[2] Balke N, Jesse S, Morozovska A N, Eliseev E, Chung D W, Kim Y, Adamczyk L, García R E, Dudney N, Kalinin S V (2010). Nat Nanotechnol.

[3] Dokukin M E, Guz N V, Gaikwad R M, Woodworth C D, Sokolov I (2011). Phys Rev Lett.

[4] Dong M, Sahin O (2011). Nat Commun.

[5] Forchheimer D, Platz D, Tholén E A, Haviland D B (2012). Phys Rev B.

[6] Garcia R, Herruzo E T (2012). Nat Nanotechnol.

[7] Jesse S, Kalinin S V, Proksch R, Baddorf A P, Rodriguez B J (2007). Nanotechnology.

[8] Martínez N F, Lozano J R, Herruzo E T, Garcia F, Richter C, Sulzbach T, Garcia R (2008). Nanotechnology.

[9] Platz D, Tholén E A, Pesen D, Haviland D B (2008). Appl Phys Lett.

[10] Proksch R (2006). Appl Phys Lett.

[11] Raman A, Trigueros S, Cartagena A, Stevenson A P Z, Susilo M, Nauman E, Contera S A (2011). Nat Nanotechnol.

[12] Sikora A, Bednarz L (2011). Cent Eur J Phys.

[13] Solares S D, Chawla G (2010). J Appl Phys.

[14] Tetard L, Passian A, Thundat T (2010). Nat Nanotechnol.

[15] Tetard L, Passian A, Venmar K T, Lynch R M, Voy B H, Shekhawat G, Dravid V P, Thundat T (2008). Nat Nanotechnol.

[16] Xu X, Melcher J, Basak S, Reifenberger R, Raman A (2009). Phys Rev Lett.

[17] Abak M K, Aktas O, Mammadov R, Gürsel I, Dâna A (2008). Appl Phys Lett.

[18] Hutter C, Platz D, Tholén E A, Hansson T H, Haviland D B (2010). Phys Rev Lett.

[19] Lozano J R, Garcia R (2008). Phys Rev Lett.

[20] Sahin O, Magonov S, Su C, Quate C F, Solgaard O (2007). Nat Nanotechnol.

[21] Sarioglu A F, Solgaard O (2008). Appl Phys Lett.

[22] Solares S D, Hölscher H (2010). Nanotechnology.

[23] Stark M, Stark R W, Heckl W M, Guckenberger R (2002). Proc Natl Acad Sci U S A.

[24] Stark R W, Naujoks N, Stemmer A (2007). Nanotechnology.

[25] Platz D, Forchheimer D, Tholén E A, Haviland D B (2013). Nat Commun.

[26] Williams J C, Solares S D (2013). Beilstein J Nanotechnol.

[27] Cronin-Golomb M, Murphy A R, Mondia J P, Kaplan D L, Omenetto F G (2012). J Polym Sci, Part B: Polym Phys.

[28] Ihalainen P, Järnström J, Määttänen A, Peltonen J (2011). Colloids Surf, A.

[29] Leung K M, Wanger G, Guo Q, Gorby Y, Southam G, Lau W M, Yang J (2011). Soft Matter.

[30] Qu M, Deng F, Kalkhoran S M, Gouldstone A, Robisson A, Van Vliet K J (2011). Soft Matter.

[31] Sahin O, Erina N (2008). Nanotechnology.

[32] Schön P, Dutta S, Shirazi M, Noordermeer J, Vancso G J (2011). J Mater Sci.

[33] Sweers K, van der Werf K, Bennink M, Subramaniam V (2011). Nanoscale Res Lett.

[34] Wang S, Zhao G (2012). Mater Lett.

[35] Li C, Vepari C, Jin H-J, Kim H J, Kaplan D L (2006). Biomaterials.

[36] Sahin O (2007). Rev Sci Instrum.

[37] Belov M, Quitoriano N J, Sharma S, Hiebert W K, Kamins T I, Evoy S (2008). J Appl Phys.

[38] Wang M, Jin H-J, Kaplan D L, Rutledge G C (2004). Macromolecules.

